# 976 Improving Communication Through Education of Initial Burn Management for Patients Awaiting Outpatient Burn Clinic Follow-up

**DOI:** 10.1093/jbcr/iraf019.507

**Published:** 2025-04-01

**Authors:** Krystle Ferreira

**Affiliations:** University of Utah Burn Trauma ICU

## Abstract

**Introduction:**

There has been a decrease in burn injuries due to public awareness and safety efforts, thereby limiting providers’ experience with assessment, management, and familiarity with the American Burn Association (ABA) referral criteria to verified burn centers. Inexperience can lead to unnecessary medical interventions, inappropriate transfers, increased costs, and adverse patient outcomes. During a review of the burn call referral process with the leadership team of an ABA-verified burn center, the staff identified a gap in communication, education, and available resources between it and its referring providers. The team prioritized the clinical problem and requested a modification of the current process to incorporate a standardized patient education handout for patients awaiting burn clinic follow-up. The previous burn call referral process utilized verbal communication of initial burn management. For patients not requiring inpatient admission, the burn provider provides care recommendations to the charge nurses, who then communicate the recommendations to the referring providers. A standardized patient education handout was created from existing evidence-based resources. A modified workflow was implemented to incorporate the education handout to be sent via fax or email.

**Methods:**

A qualitative analysis of the charge nurse group was utilized to assess perceptions and barriers. Data was collected over ten weeks from an electronic telephone intake form to evaluate the frequency and use of the handout. A post-assessment evaluation assessed the revised process’s usability, feasibility, satisfaction, and recommendations for future implications.

**Results:**

Overall compliance with the modified referral process and the utilization of the patient education handout was 49%. High satisfaction for usability (94%) and feasibility (94%) were reported by the charge nurses. One hundred percent of the stakeholders agree that the patient education handout is a valuable resource and approve of the modified process. Stakeholders intend to continue using the handout beyond the implementation of this project. Additionally, they provided suggestions for revisions, which included instructions for wound care of chemical burns, frostbite, face care, and alternative topical treatments.

**Conclusions:**

The patient education handout is a valuable resource for improving communication between the burn center and its referring providers. The data demonstrates a disconnect between the overwhelming satisfaction of the project and the stakeholder’s compliance.

**Applicability of Research to Practice:**

The available literature acknowledges provider inexperience while identifying a need for additional education and resources when managing burn patients. This project took the initiative to improve communication and education and provide resources to referring providers with efforts to improve patient care and outcomes.

**Funding for the Study:**

This project was quality improvement in nature and was not subject to external funding.

